# Available web-based teaching resources for health care professionals on screening for oral cancer

**DOI:** 10.4317/medoral.20163

**Published:** 2014-12-05

**Authors:** Pablo Varela-Centelles, Angel Insua, Juan M. Seoane-Romero, Saman Warnakulasuriya, Alexander Rapidis, Pedro Diz, Juan Seoane

**Affiliations:** 1 OMEQUI Research Group. School of Medicine and Dentristry. Santiago de Compostela University. 15782 Santiago de Compostela (A Coruña). Spain; 2 EOXI Lugo. Galician Health Service. Praza Ferrol 11. 27001 Lugo. Spain; 3 Oral Medicine, King’s College, London, UK; 4 WHO Collaborating Centre for Oral Cancer, London, UK; 5 Greek Anticancer Institute, Saint Savvas Hospital, Athens, Greece

## Abstract

Objectives: To identify websites with adequate information on oral cancer screening for healthcare professionals (HCPs) and to assess both their quality and contents. Study Design: Websites were identified using Google and HON medical professional search engines using the terms “screening for oral cancer”. The first 100 sites retrieved by each engine were analysed using the DISCERN questionnaire (reliability), the V instrument (contents on oral cancer) and further by the Flesch-Kinkaid Reading Grade Level and the Flesch Reading Ease (readability). Results: The overall rating showed minimal shortcomings in the quality of the information in the websites. The coverage and correctness of information on “visual examination” was rated as fair/good, whereas updating of contents resulted very variable (eg: 81% for visual examination and 18.2% for molecular biomarkers). These results permitted to rank the websites housing relevant information for oral cancer. Top ranking websites were affiliated to the Oral Cancer Foundation (USA), WHO Collaborating Centre for oral cancer (UK) whose webpage is entitled “Oral Cancer Education and Research”, and the Clinical Guidelines maintained by the British Columbia Cancer Agency (Canada) and the British Dental Association (UK) respectively. Conclusions: There are web-based, HCP-addressed, resources on screening for oral cancer housing heterogeneous information both in quality and contents. The use of specific evaluation tools permits the selection of reliable websites on this topic with a potential to improve the existing educational gaps among HCPs.

** Key words:**Oral cancer, early diagnosis, screening, secondary prevention, internet, teaching resources, continuous education.

## Introduction

Internet search for medical or health-related issues is very frequent, and cancer-related topics account for a substantial proportion of these searches even when significant discrepancies in terms of quality of the oncological information available on-line have been reported (1,2).

A study unveiled that on-line cancer-related contents seem to help physician-patient communication, favour shared deci-sion-making, and facilitate the setting of realistic expectations (3).

The issue of web-based information on pre cancer (potentially malignant disorders) has also been studied (4,5), and both topics (pre cancer and cancer) have scored uneven accuracy levels (6) and low quality standards (7), but these shortcomings do not seem to discourage patients. Even the reported distrust of the information provided on-line is perceived as a minor reason for not using web-based resources to find oral cancer information (8).

Healthcare professionals’ (HCP) knowledge on oral cancer is widely variable and frequently sub optimal (9). Physicians achieve important parts of their knowledge -up to 80%- informally on the Internet (10), particularly those from middle-income countries (11), mostly seeking information on diagnostic and laboratory tests. Unreliability of the web-based information is a serious threat for Internet use in clinical information seeking (12). No investigation assessing the quality of available web-based oral cancer information for HCPs could be retrieved.

Thus, the aim of this study was to identify web sites with free-access in the Internet with adequate information on oral cancer screening for HCPs and to assess both their quality and contents.

## Material and Methods

-Searching strategy

Web sites were identified in November 2013 by means of 2 search engines: Google (www.google.com) and HON medical professional (www.hon.ch/med.html), using the terms “screening for oral cancer” and the English language for the interface and operative system, without predetermined location or filters. The web sites were displayed (10 sites per page), accessed, and saved in a DVD for analysis.

The first 100 consecutive results, as sorted by the search engines, were considered for the study. Exclusion criteria were: irrelevant contents, exclusively commercial information, patient-targeted sites, duplicated web sites, forums and discussion groups, non-operative sites, and password-protected web pages.

-Evaluation procedures

The selected web sites were categorised by specialization (totally or partially related to oral cancer screening) and affiliation (nonprofit organization, commercial, university/medical centre, government) (6). We also recorded whether the web site was awarded the Health On the Net (HON) seal: a nonprofit foundation to guide lay people and HCPs to reliable health-related information on the Internet. The HON code of conduct for medical and health web sites ensures the criteria of authoritative, complementarity, privacy, attribution, justifiability, transparency, financial disclosure and advertising policy.

-Quality assessment

Quality was rated using the DISCERN questionnaire, a valid and reliable tool where each question (n=16) is scored in a 1 (poor) to 5 (good) Likert scale. Questions 1 to 8 address the reliability of the publication (trust) as a source of information (13), and includes a final item on the overall rating of the publication which was considered an outcome of this investigation. The review process was independently undertaken by two observers (IA & VP); in case of disagreement, a third reviewer (coordinator) was involved.

The information in each site was assessed using the oral cancer websites content review instrument V (14), specifically designed for analyzing oral cancer contents, which is available at www.dentalinformatics.org/tools/oral cancer/.

The study was restricted to diagnostic information, which was analyzed in terms of presence (yes/no), coverage of information, correctness of information (both in a 4-point Likert scale: good, fair, poor, not available (N/A). Each answer choice carried a number of points (good=3; fair=2; poor=1; N/A=0) (15).

The retrieved information and its correctness were evaluated according to existing evidence (15,16), and the scores (good coverture and correctness achieved for the visual examination item in the analysis of contents) were used to identify web sites housing relevant information.

-Readability assessment

The Flesch-Kinkaid Reading Grade Level (FKRGL) and the Flesch Reading Ease (FRES) were used to assess legibility of the selected web sites (17,18). These score systems are well validated for assessing the readability of English-written information (19). An online calculator program (www.readabilityformulas.com) was employed for this purpose, prior determination of its accuracy using the following readability formulas: FRE= 206.835 - (1.015 x Average number of words per sentence) - (84.6 x Average number of syllables per word); FKRGL= (0.39 x Average number of words per sentence) + (11.8 x Average number of syllables per word) - 15.59.

-Statistical analysis

Data were coded, recorded and analyzed using a statistical package (SPSS Inc, Chicago, IL, USA). A descriptive study was performed and both normality and variance homogeneity were analyzed using the Kolmogorov-Smirnov and Levene tests respectively. The independent samples t-test was used for comparing means. The confidence level chosen for all tests was 95%.

## Results

**Figure 1:**
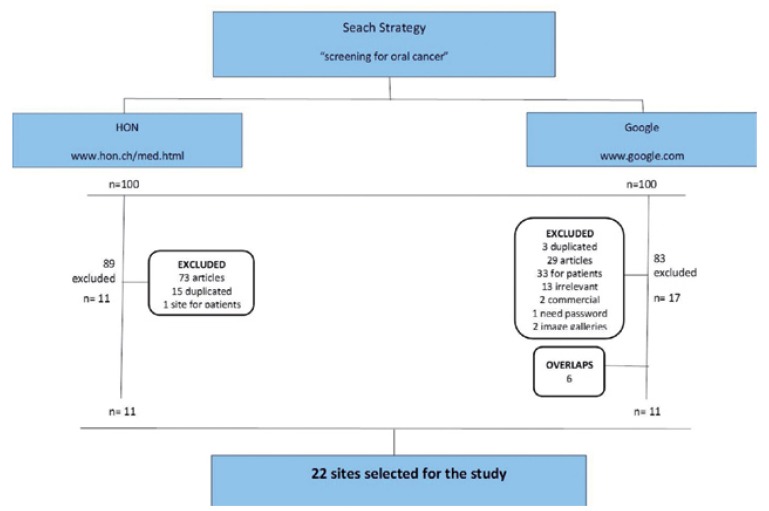
Website strategy design for the 200 accessed sites and exclusions.

Google search yielded 402,000 results in 0.18 seconds whereas HON returned 5,520 hits in 0.26 seconds. Out of the first 100 consecutive sites considered by each search engine, 83 google-search sites and 89 HON-search sites did not meet the inclusion criteria and were excluded from the study. Another 6 sites were recognized by both browsers. Finally, 22 web sites were included in the study (Fig. 1).

Chosen web pages mostly bore the seal of either government (n=10; 45.5%) or nonprofit organization (n=9; 40.9%) affiliations, with a low grade of specialization, and only 5 (22.7%) were exclusively related to oral cancer screening.

No significant differences between search engines could be disclosed in terms of contents, quality and legibility of the information ( Table 1).

**Table 1:**
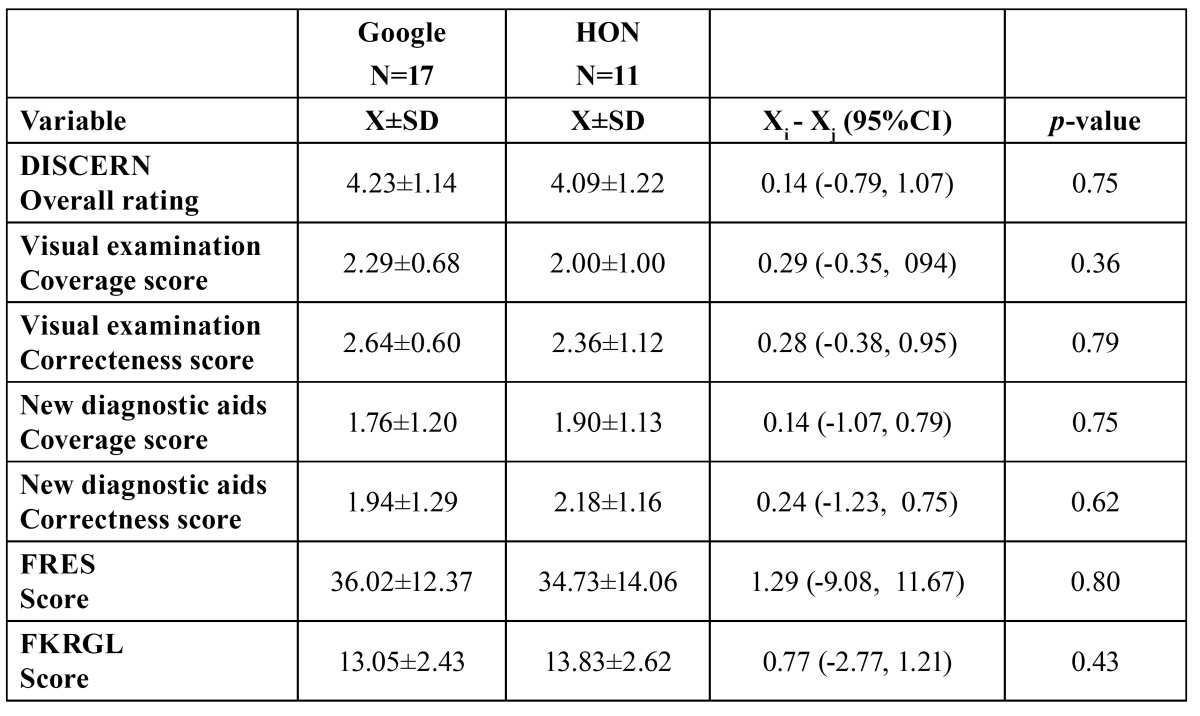
Data on contents analysis, quality and readability of the selected web-sites (Google vs HON search engine).

**Figure 2:**
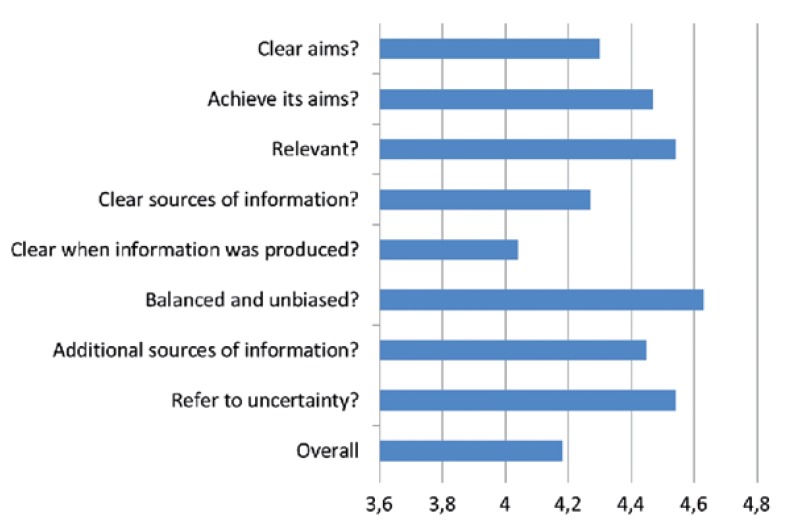
Mean quality ratings across the 22 included sites, using the validated DISCERN instrument.

The mean score for the overall rating of the publications using the DISCERN scale was 4.18±1.05, so minimal shortcomings in the quality of the information in the web sites considered in the study can be assumed (Fig. 2).

**Figure 3:**
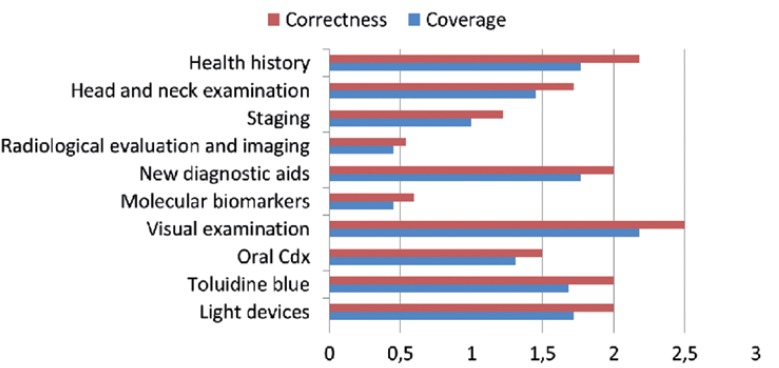
Mean Content Evaluation across the 22 included sites.

The contents assessment in terms of coverage and correctness is displayed in figure 3, where information on visual examination reached an average somewhere between “fair” and “good.”

Updating of contents resulted to be very variable among web sites, ranging between 81% (n=18) for information related to visual examination, and 18.2% (n=4) for molecular biomarkers.

The combined application of the information collected from this study has permitted us to rank web sites on coverage basis or quality ( Table 2). Top ranking sites are affiliated to (i) the Oral Cancer Foundation, (ii) the web page maintained by the WHO C-C on oral cancer entitled “Oral Cancer Education and Research”, (iii) the clinical guidelines maintained by the British Columbia Cancer Agency and (iv) the British Dental Association respectively.

**Table 2:**
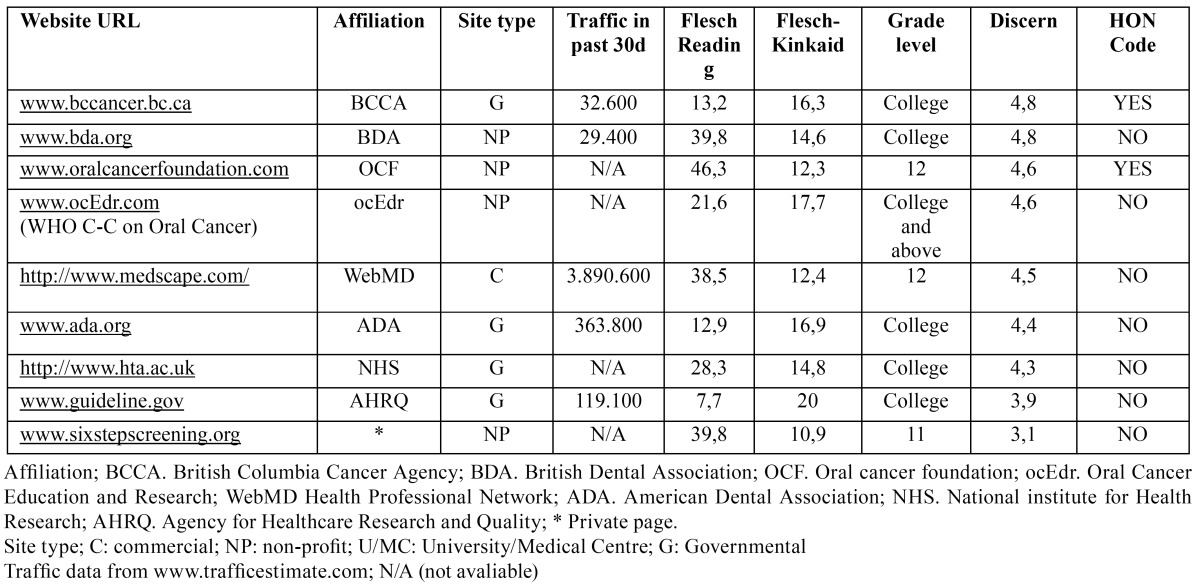
Features of the top 9 websites by Content and Quality rating.

## Discussion

There are certain shortcomings of this investigation that need to be addressed: the variations on the order on which results are listed by search engines over time may hamper reproducibility of the study. Besides, the investigation was constrained to English language web sites, thus in a global context generalization of the results is limited.

A recent meta-analysis suggested diagnostic delay is a moderate risk factor for mortality from head and neck cancer, but when the analysis was restricted to referral delay, the latter was associated to a threefold increase in mortality (20). Delayed referrals are often due to the inadequate knowledge and skills of HCPs (21), a common worldwide situation (22).

Lack of knowledge and experience are recognized as the main barriers to the provision of routine oral cancer examinations (21). Medical web sites seem to fulfill the requirements for an ideal source of medical information to minimize this problem. Lack of knowledge, issues with information technology or online sources and limited search skills have been suggested as obstacles for Internet use by HCPs (11). Conversely, new technologies like mobile equipment availability and new apps for practitioners would allow learning, teaching and practicing anywhere, anytime (23).

Patients frequently use the Internet to agree or not on consenting to surgical decision making (24), and head-neck cancer patients usually seek information on treatment and secondary effects (managing changes in swallowing and speaking), and on how to maintain their health after treatment (8,25,26). Physicians tend to use web-based resources to ease clinical decision making (24), mostly related to diagnostic work-up and therapy (26). However, both the presence of irrelevant contents and their poor reliability are paramount barriers to using the Internet for information seeking (12).

Previous reports have described a poor quality of the patient-addressed information about lichen planus, oral leukoplakia, and oral and head and neck cancers (4-7). On the other hand, our study indicates that the overall quality rate of the information about screening for oral cancer in HCP-addressed web sites reached a high standard on application of the DISCERN instrument, particularly the sites listed in the first four places, which achieved an score higher than 4.5, showing minimal shortcomings.

Despite DISCERN is a validated, widely used tool for determining the reliability of a publication, it was not designed for assessing the accuracy of the scientific contents displayed within the site (6,14).

Dental care professionals should remain vigilant for signs of oral cancer whilst performing routine oral examination in practice (15) because visual and tactile examination may result in early detection of oral carcinomas (stages I-II) (14,16); this being the reason to employ the “adequate coverage and correctness” of the information on this topic to identify relevant HCP-addressed web sites.

It is worth mentioning that although ancillary tests for oral cancer screening (eg.: toloudine blue or commercial devices based upon tissue reflectance or auto fluorescence) have not been adequately tested for primary care use (16), the web sites selected for this study offer an adequate information on this topic (Fig. 2). Only molecular biomarkers and transepithelial cytology (Oral Cdx) were poorly covered by these web pages.

To the best of our knowledge, no previous study has assessed the readability of oral cancer web sites; defined as the reading skills an individual must possess to understand a written text (17). By extracting FRES for our selection of web sites, we found the contents “difficult to read”, probably due to the technical nature of the information displayed. These contents would also be difficult for patients to understand, as the reading grade level calculated for these web sites is well above what is recommended for health-related materials for patients (6th or below) (27).

WHO has recommended involving oral-health professionals in detection and early diagnosis of oral cancer (28). Even though increasing knowledge and skills for oral cancer screening is traditionally considered as a chief educational objective related to secondary prevention, there are still gaps of knowledge among HCPs on this topic (21) which may condition effectiveness of screening for oral cancer. In this sense, web-based oral cancer resources may contribute to improve the current situation particularly by creating international cooperation networks and electronic web sites housing worldwide information for training on this topic (29). Based on an EU initiative on lifelong learning our group has been commissioned to deliver a web based learning programmer on the early detection of oral cancer for European Dentists. This can be accessed via www.oralcancerldv.org.

## Conclusion

Our study demonstrated that there are web-based, HCP-addressed, resources on screening for oral cancer housing heterogeneous information both in quality and contents. The use of specific evaluation tools permits the selection of reliable web sites on this topic with a potential to help reducing the existing educational gaps among HCPs.
